# Ammonium bio-ionic liquids based on camelina oil as potential novel agrochemicals†

**DOI:** 10.1039/c8ra03519a

**Published:** 2018-08-13

**Authors:** Juliusz Pernak, Bartosz Łęgosz, Tomasz Klejdysz, Katarzyna Marcinkowska, Jacek Rogowski, Danuta Kurasiak-Popowska, Kinga Stuper-Szablewska

**Affiliations:** Poznan Univeristy of Technology, Faculty of Chemical Technology Berdychowo 4 60-965 Poznań Poland juliusz.pernak@put.poznan.pl; Institute of Plant Protection – National Research Institute Węgorka 20 60-101 Poznań Poland; Poznan University of Life Sciences, Faculty of Agronomy and Bioengineering Dojazd 11 60-632 Poznań Poland; Poznan University of Life Sciences, Faculty of Wood Technology Wojska Polskiego 75 60-625 Poznań Poland

## Abstract

Third generation bio-ionic liquids (bio-ILs) were synthesized based on cheap and increasingly available camelina oil. The ionic liquids were obtained with high yield based on the reaction between camelina oil, which contained the following carboxylic acids: C18:3ω-3 linolenic >30%, C20:1 eicosenoic 28%, C18:2ω-6 linoleic 13%, C18:1 oleic 13%, C16:0 palmitic 4.5%, C22:1 erucic 4.5% and C18:0 stearic 2.5%, and quaternary ammonium hydroxides comprising cations such as: choline, di(hydrogenated tallow)dimethylammonium, oleylmethylbis(2-hydroxyethyl)ammonium, benzalkonium, tetradecyltrimethylammonium, tetramethylammonium and didecyldimethylammonium. The synthesized bio-ILs were characterized as high viscosity liquids which are thermally stable and their solubility in water and organic solvents depended on the type of cation. Two extreme examples of bio-ILs include the water soluble one comprising choline as the cation and the one comprising the di(hydrogenated tallow)dimethylammonium cation, which is soluble in hexane. The presented results show the importance of ammonium bio-ILs as antifeedants with a wide spectrum of activity. The tested beetles (Insecta: Coleoptera) of storage pests: grain weevil (*Sitophilus granarius* (L.)), confused flour beetle (*Tribolium confusum* Duv.) and khapra beetle (*Trogoderma granarium* Ev.) presented notable differences in terms of susceptibility to the synthesized ILs. The synthesized bio-ILs are effective adjuvants for herbicides belonging to the sylfonylurea group. They exhibited high activity despite the fact they were applied at a dose almost half that used for commercial adjuvants, which opens the era of adjuvant ILs. Aside from its use in the production of biodiesel, renewable diesel and renewable jet fuel, camelina oil is starting to become a potential resource for the production of novel agrochemicals.

## Abstract 2

Third generation bio-ionic liquids (bio-ILs) were synthesized based on cheap and increasingly available camelina oil.

## Graphical abstract

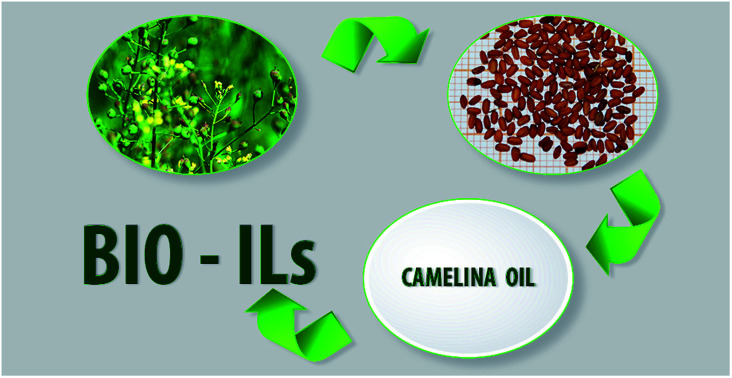

## Introduction

Ionic liquids (ILs) are a well-known group of chemical compounds, and have gained high popularity due to their unique properties. The number of possible cation–anion combinations is estimated to be over 10^18^, making them significant for numerous applications.^1^ Taking into account the physicochemical and biological properties of ILs, a general yet practical classification of these compounds into three generations was proposed in 2007.^2^ The first generation comprises ILs with specific physical properties, obtained by careful selection of appropriate cations and anions, whereas the second generation additionally considers their chemical properties. The third generation includes ILs, which simultaneously exhibit targeted biological properties as well as desired physical and chemical properties. An example of the most recent generation may be the conversion of popular drugs into ILs in order to increase their medical potential.^3–5^ However, this effect is not only limited to pharmaceuticals. In 2011 (ref. 6) a novel group of ILs called herbicidal ionic liquids (HILs) which comprise a herbicidal anion was introduced. Currently there are literature reports regarding HILs based on the following herbicides: 2,4-D,^7^ MCPA,^6^ MCPB,^8^ MCPP,^9^ dichlorprop,^10^ dicamba,^11,12^ clopyralid,^13^ fomesafen,^14^ bentazon,^15^ glyphosate,^16,17^ metsulfuron methyl,^18^ nonanoic acid^19^ and picloram.^20^ The combination of two different types of biological activity is also known to date, as exemplified by dual function ILs which function as both herbicides and growth regulators.^21^ Similar to drugs, the transformation of popular fungicides into the IL structure was also carried out.^22,23^ Additionally, some ILs were described as feeding deterrents, protecting stored crops from pests.^24^ Recently there have been examples of studies regarding the chemical modification of plant resistance inducer, benzo[1,2,3]thiadiazole-7-carboxylate (BTH).^25,26^ When natural resources are used for the synthesis of third generation ILs, they may be classified as bio-ILs.^17,27–29^ Agriculture needs a new chemical platform, for example third generation ILs, to help with crucial issues, such as resistance to herbicides.

Triglycerides present in vegetable oils are an attractive source of chemicals, mainly fatty acids, which may be a source of anions for the synthesis of ILs. Camelina is an annual oil plant, which belongs to the Brassicaceae family. Its attractiveness is associated with its resistance to negative soil and climate conditions as well as high tolerance to diseases such as *Alternaria brassicae* and pests. This allows to use low-class soils for its cultivation, which are not suitable for other crops. Additionally, camelina possesses unique agronomic traits, which include a short vegetation time and higher tendency to now sow seeds compared to flax.^30^ Consequently, the attractive production economics and minimal input requirements regarding its cultivation provide further added advantages.

During recent years there has been a growing interest in camelina due to the unique composition of camelina oil. The adaptation of camelina to the vast areas of the world, high oil content (28–40%) combined with its unique oil composition and properties make it a suitable oil source for the production of biofuels, jet fuel, bio-based products, feed, and food. Camelina oil is rich in oleic (18:1, 14–18%), linoleic, (18:2 ω-6, 15–23%), linolenic (18:3 ω-3, 28–40%), and eicosenoic (20:1, 12–17%) acid.^31^ Currently most attention is directed to the use of camelina oil for the production of biodiesel, renewable diesel and renewable jet fuel.^32^

The aim of this study was to use camelina oil as a resource for the synthesis of bio-ILs and evaluate the potential application of the obtained compounds in agricultural practices. This study was focused on the synthesis of novel bio-ILs and evaluation of their physicochemical properties, deterrent and adjuvant activity.

## Results and discussion

Camelina oil used in this study was analysed in terms of its fatty acid profile using HPLC. The presence and content of following main acids were established: C18:3ω-3 α-linolenic (ALA) >30%, C20:1 eicosenoic 28.22%, C18:2ω-6 linoleic (LA) and C18:1 oleic at 12.84% and 13.35%, respectively. The content of C16:0 palmitic (PA) and C22:1 erucic (EU) was below 5% (4.45% and 4.57%, respectively), C18:0 stearic acid was at 2.47%, C20:2 eicosadienoic at 1.86%, while C24:1 nervonic, C20:0 arachidic (ARA), C21:0 heneicosanoic and C24:0 lignoceric were present at <1% (0.98%, 0.86%, 0.61% and 0.19%, respectively). The presence of C14:0 myristic acid, C15:0 pentadecanoic acid, C15:1 pentadecenoic acid, C16:1 palmitoleic acid, C17:0 margaric acid, C17:1 10-heptadecenoic acid and C18:3ω-6 γ-linolenic acid (GLA) was not detected. The acidic value of the oil was equal to 3.12 mg NaOH/1 g, anisidine value was equal to 0.39, peroxide number was equal to 0.88, total oxidation value TOTOX was equal to 2.31, smoke point (which refers to the temperature at which the oil sample begins to smoke) was equal to 159 °C.

**Scheme 1:**
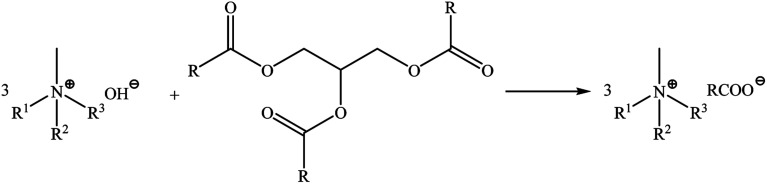
The synthesis methods of preparation of ammonium ILs with fatty acid anions isolated from camelina oil.

Ionic liquids with camelina oil anions were synthesized according to the previously described procedure.^28^ The progress and conditions of the reactions were monitored using a Mettler-Toledo EasyMax 102 system. Quaternary ammonium cations: (tetramethylammonium, tetradecyltrimethylammonium, didecyldimethylammonium, benzalkonium, choline, di(hydrogenated tallow)dimethylammonium and oleylmethylbis(2-hydroxyethyl)ammonium) with different structures were used during the syntheses in order to investigate their influence on biological properties. The cation structures did not affect the reaction time. The pH of the solutions stabilized after 20 minutes. The reaction were carried out in propan-2-ol and glycerine was the by-product (Scheme 1). All the synthesized products were obtained as high viscosity liquids at ambient temperature and did not exhibit a tendency to undergo crystallisation, which allows to classify them as ILs. In this case the anion consists of a mixture of fatty acids. The synthesized mixtures of bio-ILs (anion: C18:3ω-3 linolenic >30%, C20:1 eicosenoic 28%, C18:2ω -6 linoleic 13%, C18:1 oleic 13%, C16:0 palmitic 4.5%, C22:1 erucic 4.5% and C18:0 stearic 2.5%) will posses different physicochemical properties compared to pure ILs. This has been confirmed by recently published studies regarding binary mixtures of protic ILs derived from fatty acids, which presented a marked nonideal melting profile with the formation of solid solution.^33^

Seven bio-ILs were obtained with high yields, as presented in Table 1. This group includes novel bio-ILs which comprise *e.g.* the cholinium cation 5 as well as ammonium cations with oleyl 6 or tallow 4 substituents.

**Table 1:** Characterization of synthesized ammonium bio-ILs

IL	R^1^	R^2^	R^3^	Yield [%]
1	CH_3_	CH_3_	CH_3_	93
2	CH_3_	CH_3_	C_14_H_29_	91
3	CH_3_	C_10_H_21_	C_10_H_21_	93
4	CH_3_	Hyd. tallow^a^	Hyd. tallow^a^	88
5	CH_3_	CH_3_	C_2_H_4_OH	92
6	C_2_H_4_OH	C_2_H_4_OH	Oleyl^b^	86
7	CH_3_	CH_2_Ph	Alkyl^c^	90

a Hydrogenated tallow-mixture of saturated (97%) or unsaturated (3%) alkyl substituents-C_12_ – 1%, C_14_ – 4%, C_16_ – 31%, C_18_ – 64% in Arquad 2HT.

b Oleyl-mixture of saturated (18%) or unsaturated (82%) alkyl substituents-C_12_ – 5%, C_14_ – 1%, C_16_ – 14%, C_18_ – 80%, in Ethoquad O-12.

c Alkyl-C_12_ – 40%, C_14_ – 60%.

The structures of ILs were confirmed by analysis of NMR spectra. The signals from the anion were identified in the range from 0.87 ppm to 0.90 ppm for protons of methyl groups, while protons in the methylene group bonded with the methyl group occurred as signals from 0.96 ppm to 0.99 ppm. The signals for methylene groups in the alkyl chain occurred in range from 1.22 ppm to 1.38 ppm. Methylene protons in position *β* to the carboxylic group were noted as peaks from 1.54 ppm to 1.77 ppm. Protons bonded to carboxylic carbon atom were identified as signals between 2.75 and 2.82 ppm. Protons in position *α* to multiple bonds in alkyl chains occurred in range from 1.96 and 2.15 ppm. Protons in double bonds were noted between 5.29 and 5.41 ppm.

Protons from aliphatic substituents in the cation generated signals in ranges from 0.87 ppm to 0.90 ppm. Methylene groups in positions *γ*, *β* and *α* to the quaternary nitrogen atom generated signals in ranges 1.53–1.69 ppm, 1.96–2.15 ppm and 3.22–3.45 ppm, respectively. Methyl groups bonded directly to the charged nitrogen atom were identified as signals between 3.19 ppm and 3.38 ppm. Protons in 2-hydroxyethyl groups occurred as signals in ranges 3.56–3.71 ppm and 4.04–4.05 ppm for protons in groups in positions *α* and *β* to the quaternary nitrogen atom. Methylene group between the nitrogen atom and the aromatic ring generated a signal at 4.72 ppm. Protons in aromatic ring were identified as signals between 7.42 ppm and 7.56 ppm.

The obtained ILs were investigated for phase transitions and thermal stability. The results were presented in Table 2.

**Table 2:** Thermal characteristics of the synthesized bio-ILs

IL	*T*_g_,^a^ [°C]	*T*_c_,^b^ [°C]	*T*_m_,^c^ [°C]	*T*_onset5_,^d^ [°C]	*T*_onset50_,^e^ [°C]
1	−28.2	−14.3	−20.4; 5.6	185	222
2	−25.1	−16.3	3.2	183	220
3	−47.5	−8.2	−2.0	180	224
4	—	30.2; 47.9	6.4; 20.3	189	292
5	−45.2	−26.9	−27.1	179	220
6	−68.7	−16.7; −37.7	5.5; −17.5	182	247
7	−69.3	−19.4; −6.1	−37.5; −9.7	161	224

a Glass temperature.

b Crystallization temperature.

c Melting temperature.

d Decomposition of 5% of the sample.

e Decomposition of 50% of the sample.

The studied ILs exhibited glass transition temperatures in the range from −69.3 °C (7) to −25.1 °C (2). No glass transition was observed for di(hydrogenated tallow)dimethylammonium (4). ILs presented multiple crystallization and melting steps, which can be explained by polymorphism of natural oils. This was also noted in case of ILs derived from canola and coconut oils.^27^ Single transitions were noted for ILs with tetradecyltrimethylammonium (2) and didecyldimethylammonium (3) cations. The crystallization temperatures varied from −37.7 °C to 47.9 °C, while melting was observed between −37.5 °C and 20.3 °C. The obtained bio-ILs were thermally stable. The lowest temperature of decomposition of 5% of the sample was observed for benzalkonium IL (7), while in case of the remaining ILs the decomposition temperature ranged from 179 °C to 189 °C.

The solubility of the prepared bio-ILs was determined according to Vogel's Textbook of Practical Organic Chemistry.^34^ Representative solvents were chosen and ranked by their Snyder polarity index value in a descending order (water – 9.0, methanol – 6.6, DMSO – 6.5, acetonitrile – 6.2, acetone – 5.1, ethyl acetate – 4.4, chloroform – 4.1, toluene – 2.3, hexane – 0.0). Tests were conducted at 20 °C under ambient pressure. The results were presented in Table 3.

**Table 3:** Solubility of synthesized bio-ILs^a^

IL	Solvent								
	A	B	C	D	E	F	G	H	I
1	+	+	+	−	−	−	+		−
2	−	+	+	−	−	−	+	−	−
3	−	+	+	−	−	−	+	−	+/−
4	−	+	+	−	−	−	+	−	+/−
5	+	+	+	−	−	−	+	−	−
6	−	+	+	−	−	−	+	−	−
7	−	+	+	−	−	−	+	−	−

a A-water, B-methanol, C-DMSO, D-acetonitrile, E-acetone, F-ethyl acetate, G-chloroform, H-toluene, I-hexane; solubility: “+” – good, “+/−“ – moderate, “−“ – not visible/none.

All the synthesized bio-ILs were soluble in methanol, DMSO and chloroform. The difference in solubility in water as well as in hexane was strongly influenced by the structures of alkyl substituents. ILs with small-sized cations (1, 5) were soluble in water after the addition of 1 mL. The presence of long alkyl chains in the cation rapidly decreased the ability of water to dissolve the tested ILs. On the other hand, ILs with two long-chained saturated substituents (3, 4) caused moderate solubility in hexane. The hydroxyl (6) or aromatic (7) groups did not significantly affect the solubility of ILs in the used solvents. Similar observations were made in case of ILs with anion originating from canola and coconut oils.^28^ The obtained bio-ILs were unaffected by the contact with water and the organic solvents. During their storage in the form of aqueous, methanol or chloroform solutions no changes in their concentrations were noted.

The obtained bio-ILs were tested in terms of their antifeedant properties towards beetles of *S. granarius*, *T. confusum*, and larvae of *T. confusum* and *T. granarium*. The insects were grown on a wheat grain or whole-wheat meal diet in laboratory colonies, which were maintained at 26 ± 1 °C and 60 ± 5% relative humidity. Choice and no-choice tests for insect-feeding were conducted following a described procedure.^35^ Their activity was compared with the results obtained for azadirachtine – one of the most efficient feeding deterrent occurring in nature. Values of coefficients, *A* (absolute coefficient of deterrence) and *R* (relative coefficient of deterrence) were calculated as follows:
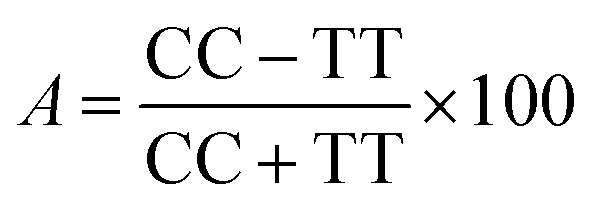

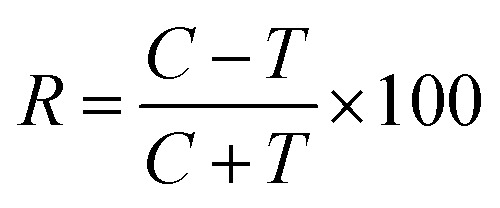
where CC is the average weight of the food consumed in the control, TT means the average weight of the food consumed in the no-choice test, while *C* and *T* express the average weights of the food consumed in the choice test. The sum of these two coefficients (*T*) explains deterrent activity: 200–151 very good, 150–101 good, 100–50 medium, <50 weak.

**Fig. 1:**
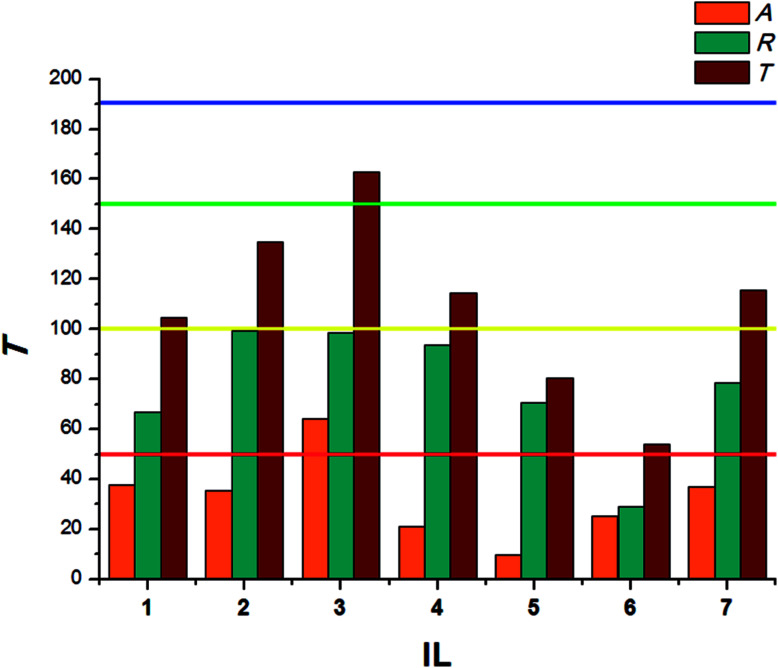
Deterrent activity of bio-ILs (1–7) towards beetles of *S. granarius*.

The results calculated for beetles of *S. granarius* were presented in Fig. 1. The blue line represents the value noted for azadirachtine.

**Fig. 2:**
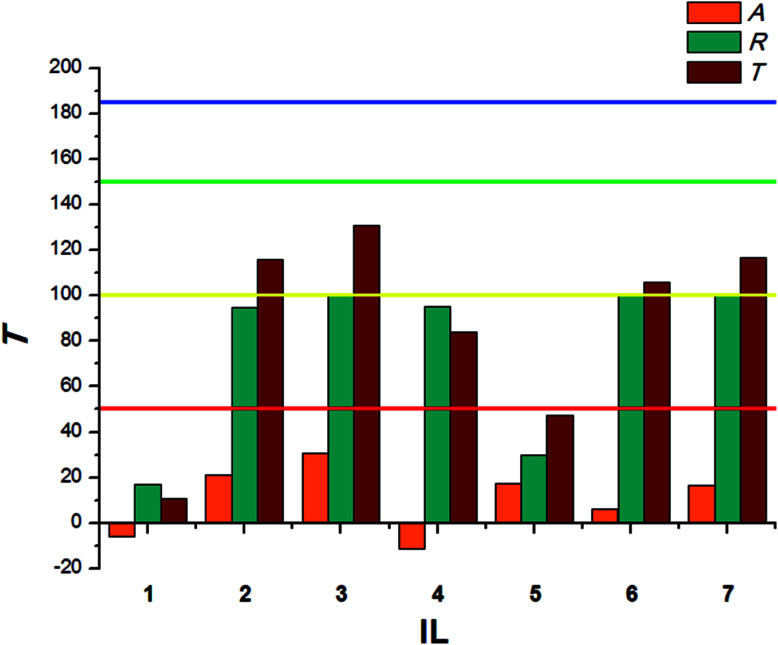
Deterrent activity of bio-ILs (1–7) towards beetles of *T. confusum*.

The presented results show moderate antifeedant properties of the synthesized bio-ILs. The highest result was reached in case of didecyldimethylammonium IL (3), which was classified as a very good deterrent (deterrent activity *T* > 150). The lowest result was observed for IL with cation comprising an oleyl substituent (6). The results obtained for *T. confusum* beetles were presented in Fig. 2.

**Fig. 3:**
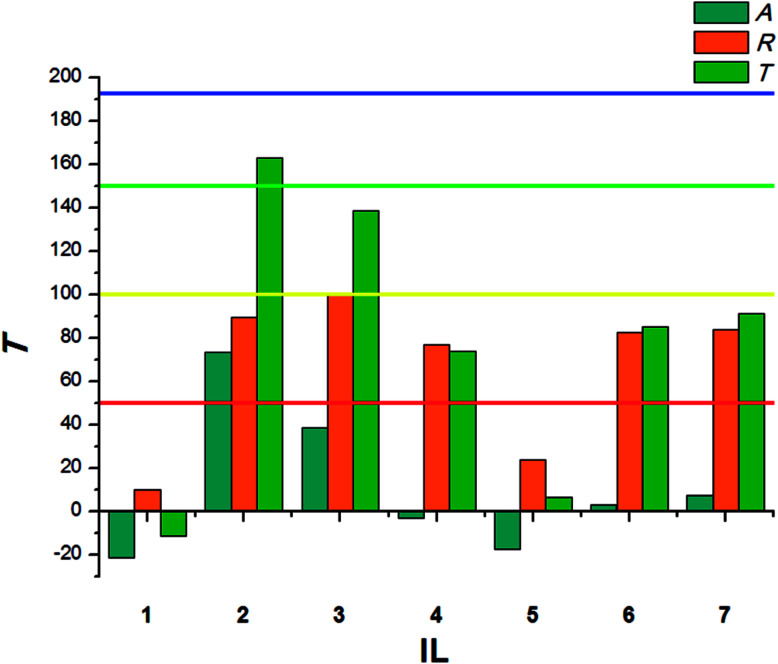
Deterrent activity of bio-ILs (1–7) towards *T. confusum* larvae.

The beetles of *T. confusum* exhibited lower susceptibility to the tested ILs when compared to *S. granarius*. Again, the highest *T* value was calculated for 3. ILs with structurally small cations (1, 5) presented the lowest activity, their values of *T* did not exceed 50. The results calculated for larvae of *T. confusum* were presented in Fig. 3.

**Fig. 4:**
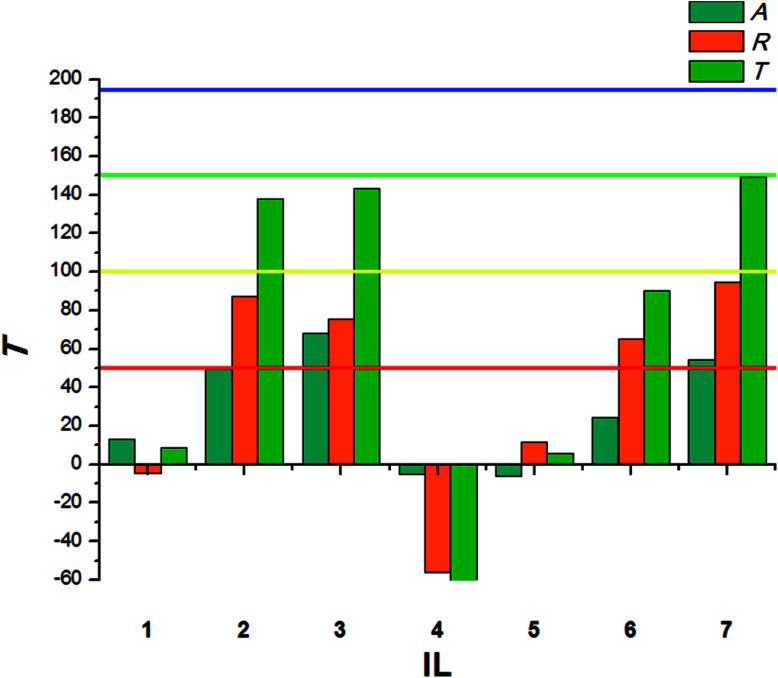
Deterrent activity of bio-ILs (1–7) towards *T. granarium*.

Within the same species, larvae present significantly lower sensitivity to the tested ILs. The highest *T* values were calculated for ILs with a long alkyl substituent in the cation – tetradecyltrimethylammonium (2) and didecyldimethylammonium (3). Small-sized cations acted as attractants, causing the larvae to preferably consume food covered with tested ILs in the no-choice test. The antifeedant activity of the synthesized bio-ILs towards larvae of *T. granarium* was presented in Fig. 4.

Similar observations were made in case of *T. granarium* larvae. Surprisingly, the lowest activity was established for the IL with hydrogenated tallow cation (4), which comprises two long saturated alkyl chains. Instead of preventing, the IL promoted the consumption of covered food in both choice and no-choice tests. On the other hand, ILs with a single tetradecyl (2) or two dodecyl (3) substituents with similar structures presented very good antifeedant properties. The difference in the obtained results may be caused by specific composition of substituents in the cation, which naturally occur in animal fat. This may make IL 4 a potentially attractive source of energy.

*T. granarium* is mainly known as a pest in crop storage areas which has adapted to the uptake of plant food. Nevertheless, the notable majority of the remaining *Trogoderma* species, 150 of which were described all over the world,^36^ feeds on animal-based food. In most cases this includes dried leathers and animal carcasses in the state of advanced decay, when the skeleton is covered only by skin, tendons and other poorly biodegradable body parts. The larvae of the *Trogoderma* species may also feed on feathers and hair. The attractiveness of the IL which contained a cation with a substituent based on animal fat to *T. granarium* may indicate the presence of primal substrate preferences of this species.

The presented results show the importance of ammonium bio-ILs as antifeedants with a wide spectrum of activity. The tested organisms presented large differences in susceptibility to the synthesized bio-ILs. The highest *T* values were calculated for ILs with long saturated substituents (2, 3), making them the most universal among the obtained compounds. In our previous studies, the best antifeedant parameters were also exhibited by didecyldimethylammonium ILs, however, as shown in the described results, the origin of anions used also plays a significant role in the final antifeedant effectiveness of the IL.^27^ The lower sensitivity to the tested ILs presented by larvae may be caused by higher amounts of food consumed needed for development.

The first published report regarding bio-ILs as adjuvants for sulfonylurea herbicides prompted us to investigate the synthesized ILs based on the cheap and increasingly available camelina oil as potential adjuvants of popular herbicides.^37^ Herbicide treatments consisted of nicosulfuron (Henik 50 SG) and ILs with fatty acid anions isolated from camelina oil or fatty acid methyl esters (Actirob 842 EC) as reference adjuvant tested against common lambsquarters, cornflower and silky bent grass. The results were given as reduction in fresh weight of plants in Table 4.

**Table 4:** Fresh weights reduction of plants treated with nicosulfuron at 30 g ai per ha with and without adjuvants at three weeks after application

Tank-mix partner	Fresh weight reduction^a^ [%]					
	Common lambsquarters		Cornflower		Silky bent grass	
1	85	A	74	ab	95	a
2	84	A	81	ab	93	a
3	89	A	85	a	91	a
4	89	A	83	ab	93	a
5	82	A	72	b	88	a
6	90	A	82	ab	90	a
7	88	A	81	ab	89	a
Actirob	89	A	82	ab	91	a
None	3	B	8	c	0	b
HSD	26.51		12.18		29.77	

a Values follow by the same letter in the same column are not significantly different (*P* = 0.05).

Nicosulfuron applied alone at 30 g ha^−1^ did not demonstrate herbicidal activity. The addition of adjuvants notably improved the herbicidal efficacy in all treatments. Results obtained for common lambsquaters and silky bent grass showed significant differences only in case of combinations with and without adjuvants. In these cases bio-ILs with fatty acid anions isolated from camelina oil showed similar efficacy as the commercial adjuvant and a level of reached weed control above 80%. Slightly different situation was observed in case of cornflower. Among combinations with adjuvants, the lowest efficacy was demonstrated by IL 5, whereas the highest by IL 3. Bioassay results show that ILs 1–7 were as effective as the commercial adjuvant in terms of improving the herbicidal activity of nicosulfuron. It is worth mentioning that the high efficacy of ILs was obtained using doses lower by approx. half compared to the adjuvant available on the market (Actirob 842 EC). Research indicated that ILs based on vegetable oils can be used as effective adjuvants for herbicides belonging to the sulfonylurea group.

## Conclusions

We confirmed that camelina oil is a good source of anions for the synthesis of bio-ILs. The reaction between camelina oil and quaternary ammonium hydroxides in propan-2-ol occurs rapidly with a high yield and glycerine is the by-product. The obtained ILs are thermally stable, high viscosity liquids, unaffected by the contact with air and organic solvents.

As presented in the framework of the study, it is possible to obtain bio-ILs, which are soluble in water as well as strongly hydrophobic solvents. ILs with fatty acid anions isolated from camelina oil and cations comprising two decyl or hydrogenated tallow substituents are soluble in hexane.

The synthesized ILs displayed deterrent activity with a strong influence of the cation type. Very good deterrent activity was noted for bio-ILs with the tetradecyltrimethylammonium and didecyldimethylammonium cations. The observed deterrent activity ranging from a lack of effect to excellent action results from the cation structure, nevertheless, the type of anion also contributes to such a strong diversity of activity. The presented results show the importance of ammonium bio-ILs as antifeedants with a wide spectrum of activity. At the same time, the studied bio-ILs are effective adjuvants for herbicides belonging to the sulfonylurea group. They exhibited high efficacy despite the fact that they were applied at a dose which was lower by approx. half compared to that used in commercial adjuvants. The excellent results obtained in the framework of this study open a new era of adjuvant ILs.

In summary, it is worth emphasizing that third generation bio-ILs were synthesized from a cheap and increasingly available camelina oil. Apart from the production of biodiesel, renewable diesel and renewable jet fuel, camelina oil is also a potential resource for the production of novel agrochemicals.

## Conflicts of interest

There are no conflicts to declare.

## Supplementary Material

RA-008-C8RA03519A-s001

## References

[cit1] Holbrey J. D., Seddon K. R. (1999). Ionic Liquids. Clean Technol. Environ. Policy.

[cit2] Hough W. L., Smiglak M., Rodriguez H., Swatloski R. P., Spear S. K., Daly D. T., Pernak J., Grisel J. E., Carliss R. D., Soutullo D. M., Davis J. H., Rogers R. D. (2007). The third evolution of ionic liquids: active pharmaceutical ingredients. New J. Chem..

[cit3] Bica K., Rogers R. D. (2010). Confused ionic liquid ions - A “liquification” and dosage strategy for pharmaceutically active salts. Chem. Commun..

[cit4] Shamshina J. L., Barber P. S., Rogers R. D. (2013). Ionic liquids in drug delivery. Expert Opin. Drug Delivery.

[cit5] Shamshina J. L., Kelley S. P., Gurau G., Rogers R. D. (2015). Develop ionic liquid drugs. Nature.

[cit6] Pernak J., Syguda A., Janiszewska D., Materna K., Praczyk T. (2011). Ionic liquids with herbicidal anions. Tetrahedron.

[cit7] Praczyk T., Kardasz P., Jakubiak E., Syguda A., Materna K., Pernak J. (2012). Herbicidal ionic liquids with 2,4-D. Weed Sci..

[cit8] Pernak J., Niemczak M., Materna K., Żelechowski K., Marcinkowska K., Praczyk T. (2016). Synthesis, properties and evaluation of biological activity of herbicidal ionic liquids with 4-(4-chloro-2-methylphenoxy)butanoate anion. RSC Adv..

[cit9] Niemczak M., Giszter R., Czerniak K., Marcinkowska K., Walkiewicz F. (2015). Bis(ammonium) ionic liquids with herbicidal anions. RSC Adv..

[cit10] Niemczak M., Biedziak A., Czerniak K., Marcinkowska K. (2017). Preparation and characterization of new ionic liquid forms od 2,4-DP herbicide. Tetrahedron.

[cit11] Cojocaru O. A., Shamshina J. L., Gurau G., Syguda A., Praczyk T., Pernak J., Rogers R. D. (2013). Ionic liquid forms of the herbicide dicamba with increased efficacy and reduced volatility. Green Chem..

[cit12] Pernak J., Giszter R., Biedziak A., Niemczak M., Olszewski R., Marcinkowska K., Praczyk T. (2017). Alkyl(C_16_, C_18_, C_22_)trimethylammonium-Based Herbicidal Ionic Liquids. J. Agric. Food Chem..

[cit13] Zhu J., Ding G., Liu Y., Wang B., Zhang W., Guo M., Geng Q., Cao Y. (2015). Ionic liquid forms of clopyralid with increased efficacy against weeds and reduced leaching from soils. Chem. Eng. J..

[cit14] Ding G., Liu Y., Wang B., Punyapitak D., Guo M., Duan Y., Li J., Cao Y. (2014). Preparation and characterization of fomesafen ionic liquids for reducing the risk to the aquatic environment. New J. Chem..

[cit15] Wang B., Ding G., Zhu J., Zhang W., Guo M., Geng Q., Gou D., Cao Y. (2015). Development of novel ionic liquids based on
bentazone. Tetrahedron.

[cit16] Pernak J., Niemczak M., Giszter R., Shamshina J. L., Gurau G., Cojocaru O. A., Praczyk T., Marcinkowska K., Rogers R. D. (2014). Glyphosate-Based Herbicidal Ionic Liquids with Increased Efficacy. ACS Sustainable Chem. Eng..

[cit17] Choudhary H., Pernak J., Shamshina J., Niemczak M., Giszter R., Chrzanowski Ł., Praczyk T., Marcinkowska K., Cojocaru O. A., Rogers R. D. (2017). Two Herbicides in a Single Compound: Double Salt Herbicidal Ionic Liquids Exemplified with Glyphosate, Dicamba, and MCPA. ACS Sustainable Chem. Eng..

[cit18] Pernak J., Niemczak M., Shamshina J. L., Gurau G., Głowacki G., Praczyk T., Marcinkowska K., Rogers R. D. (2015). Metsulfuron-methyl-based herbicidal ionic liquids. J. Agric. Food Chem..

[cit19] Pernak J., Czerniak K., Niemczak M., Ławniczak Ł., Kaczmarek D. K., Borkowski A., Praczyk T. (2018). Bioherbicidal ionic liquids. ACS Sustainable Chem. Eng..

[cit20] Tang G., Wang B., Ding G., Zhang W., Liang Y., Fan C., Dong H., Yang J., Kong D., Cao Y. (2018). Developing ionic liquid forms of picloram with reduced negative effects on the aquatic environment. Sci. Total Environ..

[cit21] Pernak J., Niemczak M., Zakrocka K., Praczyk T. (2013). Herbicidal ionic liquid with dual-function. Tetrahedron.

[cit22] Pernak J., Markiewicz B., Łęgosz B., Walkiewicz F., Gwiazdowski R., Praczyk T. (2015). Known triazole fungicides – a new trick. RSC Adv..

[cit23] Bica K., Cooke L. R., Nugent P., Rijiksen C., Rogers R. D. (2011). Toxic on purpose: ionic liquid fungicides as combinatorial crop protecting agents. Green Chem..

[cit24] Markiewicz B., Sznajdrowska A., Chrzanowski Ł., Ławniczak Ł., Zgoła-Grześkowiak A., Kubiak K., Nawrot J., Pernak J. (2014). Ionic liquids with a theophyllinate anion. New J. Chem..

[cit25] Śmiglak M., Kukawka R., Lewandowski P., Budziszewska M., Obrępalska-Stęplowska A., Krawczyk K., Zwolińska A., Pospieszny H. (2016). New Dual Functional Salts Based on Cationic Derivative of Plant Resistance Inducer—Benzo[1.2.3]thiadiazole-7-carbothioic Acid, S-Methyl Ester. ACS Sustainable Chem. Eng..

[cit26] Śmiglak M., Lewandowski P., Kukawka R., Budziszewska M., Krawczyk K., Obrępalska-Stępłowska A., Pospieszny H. (2017). Dual Functional Salts of Benzo[1.2.3]thiadiazole-7-carboxylates as a Highly Efficient Weapon Against Viral Plant Diseases. ACS Sustainable Chem. Eng..

[cit27] Pernak J., Łęgosz B., Walkiewicz F., Klejdysz T., Borkowski A., Chrzanowski Ł. (2015). Ammonium ionic liquids with anions of natural origin. RSC Adv..

[cit28] Klejdysz T., Łęgosz B., Czuryszkiewicz D., Czerniak K., Pernak J. (2016). Bio-based ionic liquids with abietate anion. ACS Sustainable Chem. Eng..

[cit29] Pernak J., Czerniak K., Biedziak A., Marcinkowska K., Praczyk T., Erfurt K., Chrobok A. (2016). Herbicidal ionic liquids derived from renewable sources. RSC Adv..

[cit30] Wysocki D. J., Chastain T. G., Schillinger W. F., Guy S. O., Karow R. S. (2013). Camelina: seed yield response to applied nitrogen and sulfur. Field Crops Res..

[cit31] Popa A., Jurcoane S., Dumitriu B. (2017). *Camelina sativa* oil - A Review. Scientific Bulletin Series F. Biotechnologies.

[cit32] Berti M., Gesch R., Eynck C., Anderson J., Cermak S. (2016). Camelina uses, genetics, genomics, production, and management. Ind. Crops Prod..

[cit33] Toledo Hijo A. A. C., Maximo G. J., Cunha R. L., Fonseca F. H. S., Cardoso L. P., Pereira J. F. B., Costa M. C., Batista E. A. C., Meirelles A. J. A. (2018). Phase equilibrium and physical properties of biobased ionic liquid mixtures. Phys. Chem. Chem. Phys..

[cit34] Furniss B. S. , Hannaford A. J., Smith P. W. G. and Tatchell A. R., Vogel's Textbook of Practical Organic Chemistry, Longman, Harlow, 5 edn, 1989

[cit35] Szczepanik M., Obara R., Szumny A., Gabryś B., Halarewicz-Pacan A., Nawrot J., Wawrzeńczyk C. (2005). Synthesis and insect antifeedant activity of precocene derivatives with lactone moiety. J. Agric. Food Chem..

[cit36] Háva J. , World Catalogue of Insects. Vol. 13. Dermestidae (Coleoptera), Brill, Leiden, Boston, 2015

[cit37] Marcinkowska K., Praczyk T., Łęgosz B., Biedziak A., Pernak J. (2018). Bio-ionic liquids as adjuvants for sulfonylurea herbicides. Weed Sci..

