# Supplementary material for: Ammonium bio-ionic liquids based on camelina oil as potential novel agrochemicals
Source: RSC Adv. 2018 Aug 13;8(50):28676–83. doi: 10.1039/c8ra03519a (PMC9084345; doi:10.1039/c8ra03519a)
Supplement: RA-008-C8RA03519A-s001 [file RA-008-C8RA03519A-s001.pdf]

Electronic Supplementary Material (ESI) for RSC Advances.
This journal is © The Royal Society of Chemistry 2018

Electronic Supplementary Information

Ammonium bio-ionic liquids based on camelina oil as potential novel agrochemicals

Juliusz Pernak[a], Bartosz Łęgosz[a], Tomasz Klejdysz[b], Katarzyna Marcinkowska[b], Jacek Rogowski[b], Danuta Kurasiak-Popowska[c], Kinga Stuper-Szablewska[d]

[a] Poznan Univeristy of Technology, Faculty of Chemical Technology, Berdychowo 4, 60-965 Poznań, Poland
[b] Institute of Plant Protection – National Research Institute, Węgorka 20, 60-101 Poznań, Poland
[c] Poznan University of Life Sciences, Faculty of Agronomy and Bioengineering, Dojazd 11, 60-632 Poznań, Poland
[d] Poznan University of Life Sciences, Faculty of Wood Technology, Wojska Polskiego 75, 60-625 Poznań, Poland

Materials

Tetramethylammonium hydroxide (25% in water), choline hydroxide (46% in water), tetradecyltrimethylammonium (min. 98%), didecyldimethylammonium (50% in water) and benzalkonium (min. 95%) chlorides were purchased in Merck and used as obtained. Di(hydrogenated tallow)dimethylammonium (Arquad 2HT, 75%) and oleylmethylbis(2-hydroxyethyl)ammonium chlorides (Ethoquad 0/12, 75%) were delivered by AkzoNobel. Potassium hydroxide and all solvents were obtained from Avantor Performance Materials.

The spring variety of *Camelina sativa* ‘Omega’, which was bred at the Department of Plant Genetics and Breeding, Poznan University of Life Sciences, was registered in the National Plant Breeders’ Rights in Poland in 2013.

Oil from camelina seeds was obtained using constant extraction in a Soxhlet apparatus in 3 repetitions. 50 g of seeds were ground using a WŻ-50 laboratory mill. They were placed on a filter paper and covered with cotton wool. The extraction in the Soxhlet apparatus was conducted for 3 hours using chloroform. The solvent was then evaporated and the oil was dried using calcium chloride.

Analysis of FAME

Samples containing 1 mL of oil were placed into 17 mL culture tubes, suspended in 2 mL of methanol, treated with 0.5 mL of 2M aqueous sodium hydroxide, and tightly sealed. The culture tubes were then placed within 250-mL plastic bottles, tightly sealed and placed inside a microwave oven (Model AVM 401/1WH; Whirlpool, Sweden) operating at 2450 MHz and

900 W maximum output. Samples were irradiated (370 W) for 20 s and, after approx. 5 min, for an additional 20 s. After 15 min. The contents of the culture tubes were neutralised with 1M aqueous hydrochloric acid; 2 mL MeOH were added and extraction with pentane (3-4 mL) was carried out within the culture tubes. The combined pentane extracts were evaporated to dryness in a nitrogen stream. In the next step, the extracts were methylated using a mixture of anhydrous methanol and sulfuric acid (1:5, v/v). The extract containing lipids was added with 0.5 mL of methanol followed by an addition of a 0.15-mL methanol/sulfuric acid mixture (1:5, v/v). The samples were heated at 70 ºC for 15 min. After the solution had been cooled 0.5 mL of n-hexane was added, followed by the addition of sufficient water to form two layers. The upper hexane layer was removed and analysed using a gas chromatograph (Agilent 5890 II) equipped with a flame ionization detector, fitted with a Supelcowax 10 column (30 m × 0.25 mm I.D., 0.25 mm film thickness). The injector and detector temperatures were 220 ºC and 240 ºC, respectively. The column temperature was programmed to increase from 60 ºC to 240 ºC at a rate of 110 ºC/min. Peaks were identified by comparing the sample peak retention times with those of known methylated fatty acid compounds.[1]

Analysis of oil parameters

The characteristic values were determined according to the following ISO standard methods: acid value – ISO 1242:2007, peroxide value expressed in miliequivalents of active oxygen/kg of oil – ISO 3960:2007, anisidine value – ISO 6885:2006. The total oxidation (Totox) value was calculated based on the peroxide and *p*-anisidine values using the following equation.[2] The smoke point of the oils was determined according to the method outlined by Nielsen.[3]

Synthesis

ILs with camelina oil anions were synthesized according to previously described procedure.[4] The progress and conditions of the reactions was monitored using a Mettler-Toledo® EasyMax™ 102 system.

*ILs with tetremethylammonium (**1**) and choline (**5**) cation*

0.03 mol of tetramethylammonium or choline hydroxide in water was placed in reactor and 50 mL of 2-propanol was added. The solution was heated to 80 ºC and 0.01 mol of camelina oil was added. The reagents were heated under reflux until the pH of the system reached a constant value. The solvent was then evaporated under reduced pressure at 60 °C, after which the content of the flask was dissolved in 15 mL of distilled water, placed in a separator and washed three times with 10 mL of diethyl ether to remove any unreacted triglyceride. After separation of the phases, the aqueous layer was placed in a flask, and water was evaporated

under vacuum. In the final step, the products were dried under reduced pressure (10 kPa) at 60 °C for 24 hours.

*ILs with long alkyl substituents (**2**-**4**, **6**, **7**)*

0.03 mol of quaternary ammonium chloride was dissolved in 50 mL of 2-propanol and then stoichiometric amount of potassium hydroxide in 2-propanol was added. The reagents were mixed under ambient conditions for 15 minutes. The precipitated inorganic salt was filtered off and 0.01 mol of camelina oil was added to solution of quaternary hydroxide. The reagents were mixed under reflux until pH of the mixture reached constant value. The products were separated and purified as described above.

NMR spectra

The NMR spectra were recorded using a Mercury Gemini 300 spectrometer with TMS as the internal standard operating at 300 MHz for 1H NMR spectra and 75 MHz for 13C NMR spectra.

Tetramethylammonium fatty acid anions isolated from camelina oil (**1**)

1H NMR (CDCl3) δ ppm: 0.87-0.89 (m, 3H); 0.96-0.99 (m, 2H); 1.24-1.38 (m, 16H); 1.74-1.78 (m, 2H); 1.99-2.12 (m, 4H); 2.75-2.82 (m, 2H); 3.38 (s, 12H); 5.30-5.40 (m, 4H).

13C NMR (CDCl3) δ ppm: 14.0; 20.4; 22.6; 25.4; 27.1; 29.2; 29.3; 29.4; 29.5; 29.6; 29.7; 29.9; 30.0; 31.8; 39.3; 55.5; 127.0; 127.5; 127.8; 128.2; 129.8; 130.1; 130.3; 131.9; 179.7.

Tetradecyltrimethylammonium fatty acid anions isolated from camelina oil (**2**)

1H NMR (CDCl3) δ ppm: 0.87-0.90 (t, J = 14 Hz, 6H); 0.96-0.99 (t, J = 15 Hz, 2H); 1.22-1.37 (m, 36H); 1.52-1.54 (m, 2H); 1.69-1.74 (m, 2H); 1.99-2.02 (m, 2H); 2.03-2.06 (m, 2H); 2.08-2.11 (m, 2H); 2.76-2.82 (m, 2H); 3.34 (s, 9H); 3.41-3.44 (m, 2H); 5.29-5.40 (m, 4H).

13C NMR (CDCl3) δ ppm: 14.0; 20.4; 21.7; 22.6; 23.0; 25.3; 25.4; 25.5; 26.2; 26.7; 27.0; 29.2; 29.3; 29.4; 29.5; 29.6; 29.7; 30.0; 31.8; 38.9; 53.1; 127.0; 127.5; 127.8; 128.2; 129.7; 130.0; 130.2; 131.8; 180.2.

Didecyldimethylammonium fatty acid anions isolated from camelina oil (**3**)

1H NMR (CDCl3) δ ppm: 0.87-0.90 (m, 9H); 0.96-0.99 (t, J = 14 Hz, 2H); 1.26-1.34 (m, 40H); 1.53-1.56 (m, 4H); 1.64-1.66 (m, 2H); 1.99-2.01 (m, 2H); 2.03-2.07 (m, 2H); 2.08-2.12 (m, 4H); 2.76-2.82 (m, 2H); 3.24 (s, 6H); 3.28-3.30 (m, 4H); 5.30-5.40 (m, 4H).

13C NMR (CDCl3) δ ppm: 14.0; 20.4; 21.7; 22.5; 25.5; 26.2; 27.0; 27.2; 29.1; 29.2; 29.3; 29.4; 29.5; 29.6; 29.7; 29.9; 30.0; 31.7; 38.8; 51.4; 127.0; 127.5; 127.8; 128.1; 129.7; 130.1; 130.2; 131.8; 180.2.

Di(hydrogenated tallow)dimethylammonium fatty acid anions isolated from camelina oil (**4**)

1H NMR (CDCl3) δ ppm: 0.87-0.89 (t, *J* = 14 Hz, 9H); 0.96-0.99 (t, *J* = 15 Hz, 2H); 1.26-1.36 (m, 76H); 1.58-1.60 (m, 2H); 1.64-1.69 (m, 4H); 1.99-2.09 (m, 2H); 2.11-2.15 (m, 2H); 2.75-2.82 (m, 2H); 3.33 (s, 6H); 3.38-3.41 (m, 4H); 5.29-5.41 (m, 4H).

13C NMR (CDCl3) δ ppm: 14.0; 20.4; 21.7; 22.4; 22.5; 22.6; 25.3; 25.4; 26.2; 27.1; 27.2; 29.1; 29.2; 29.3; 29.5; 29.6; 29.7; 30.0; 30.1; 31.8; 39.2; 51.1; 127.0; 127.4; 127.7; 127.8; 128.1; 128.2; 129.7; 129.8; 130.1; 130.2; 130.3; 131.8; 179.7.

Choline fatty acid anions isolated from camelina oil (**5**)

1H NMR (CDCl3) δ ppm: 0.87-0.90 (m, 3H); 0.96-0.99 (m, 2H); 1.22-1.34 (m, 16H); 1.53-1,57 (m, 2H); 2.00-2.12 (m, 2H); 2.76-2.82 (m, 2H); 3.31 (s, 9H); 3.64-3.65 (m, 2H); 4.03-4.07 (m, 2H); 5.30-5.40 (m, 4H).

13C NMR (CDCl3) δ ppm: 14.0; 14.2; 20.5; 21.8; 22.5; 24.9; 25.4; 25.5; 26.9; 27.2; 28.9; 29.2; 29.3; 29.5; 29.6; 29.7; 29.8; 29.9; 30.0; 31.4; 31.8; 38.9; 54.4; 55.9; 127.0; 127.6; 127.8; 127.9; 128.2; 129.8; 130.0; 130.1; 130.2; 131.9; 180.4.

Bis(2-hydroxyethyl)methyloleylammonium fatty acid anions isolated from camelina oil (**6**)

1H NMR (CDCl3) δ ppm: 0.87-0.89 (m, 9H); 0.96-0.99 (m, 2H); 1.26-1.37 (m, 38H); 1.59-1.62 (m, 2H); 1.69-1.71 (m, 2H); 1.96-2.09 (m, 8H); 2.79-2.82 (m, 2H); 3.24 (s, 3H); 3.42-3.45 (m, 2H); 3.56-3.71 (m, 4H); 4.03-4.05 (m, 4H); 5.30-5.40 (m, 6H).

13C NMR (CDCl3) δ ppm: 14.0; 20.5; 21.8; 22.6; 25.0; 25.5; 25.6; 26.4; 26.8; 27.1; 27.2; 27.3; 28.9; 29.0; 29.1; 29.3; 29.4; 29.5; 29.7; 29.8; 29.9; 31.9; 34.7; 38.6; 41.5; 50.2; 55.6; 57.8; 127.1; 127.6; 127.7; 127.9; 128.0; 128.2; 129.5; 129.7; 129.8; 130.0; 130.2; 130.3; 131.9; 181.1.

Benzalkonium fatty acid anions isolated from camelina oil (**7**)

1H NMR (CDCl3) δ ppm: 0.87-0.90 (m, 6H); 0.96-0.99 (m, 2H); 1.22-1.37 (m, 36H); 1.54-1.56 (m, 2H); 1.74-1.76 (m, 2H); 1.98-2.12 (m, 6H); 2.76-2.81 (m, 2H); 3.19 (s, 6H); 3.22-3.24 (m, 2H); 4.72 (s, 2H); 5.33-5.40 (m, 4H); 7.42-7.44 (m, 3H); 7.54-7.56 (m, 2H).

13C NMR (CDCl3) δ ppm: 14.0; 20.5; 21.8; 22.6; 22.7; 24.9; 25.2; 25.5; 26.3; 26.9; 27.2; 29.2; 29.3; 29.5; 29.6; 29.7; 29.9; 31.8; 38.9; 50.1; 126.9; 127.0; 127.5; 127.6; 127.8; 127.9; 128.2; 129.1; 129.7; 129.8; 130.1; 130.3; 130.4; 131.9; 132.9; 180.3.

Thermal analysis

Thermal transition temperatures were determined by DSC using a Mettler-Toledo Stare DSC1 (Leicester, UK) unit, under nitrogen. ILs (between 5 mg and 15 mg) were placed in aluminum pans and heated from 25 °C to 120 °C at a heating rate of 10 °C min^{-1}, cooled with an intracooler at a cooling rate of 10 °C min^{-1} to -100 °C, and then heated again to 120 °C.

Thermogravimetric analysis was performed using a Mettler-Toledo Stare TGA/DSC1 unit (Leicester, UK), under nitrogen. ILs (between 2 mg and 10 mg) were placed in aluminum pans and heated from 30 °C o 450 °C at a heating rate of 10 °C min^{-1}.

Solubility

The solubility of the prepared ILs was determined according to Vogel's Textbook of Practical Organic Chemistry.[5] Representative solvents were chosen and ranked by their Snyder polarity index value in descending order (water – 9.0, methanol – 6.6, DMSO – 6.5, acetonitrile – 6.2, acetone – 5.1, ethyl acetate – 4.4, chloroform – 4.1, toluene – 2.3, hexane – 0.0). Tests were conducted at 20 °C under ambient pressure. The term 'complete solubility' refers to ILs, which were dissolved (0.1 g of IL) in 1 mL of the solvent, while the term 'limited solubility' means that 1 g of IL was dissolved in 3 mL of the solvent. The term 'insoluble' was used to describe no solubility of 0.1 g of IL in 3 mL of the solvent.

Deterrent activity

The bioassay experiments were conducted with adulys of *Sitophilus granarius*, and adults of *Tribolium confusum* Duv., and larve *Trogoderma granarium*. The insects were grown on a wheat grain or whole-wheat meal diet in laboratory colonies, which were maintained at 26 ± 1 ˚C and 60 ± 5% relative humidity. Choice and no-choice tests for insect-feeding were conducted following a previously described procedure.[6] Wheat wafers discs (1 cm in diameter, 1 mm thick) were saturated by dipping either in methanol only (control) or in a solution of the studied ILs (1%) in methanol to be tested. After evaporation of the solvent (30 min of air-drying) the wafers were weighted and offered to the insects in plastic boxes as the sole food source for 5 days. The feeding of insects was recorded under three sets of conditions: (1) on two control discs (*CC*), (2) on a choice between one treated disc (*T*) and one control disc (*C*; choice test), and (3) on two treated discs (*TT*; no-choice test). Each of the three experiments was repeated five times with 3 adults of *S. granarius*, 20 adults and 10 larvae of *T. confusum* and 10 larvae of *T. granarium.* The number of individual insects depended on the intensity of their food consumption. The adults used for experiments were unsexed, 7-10 days old, and the larvae were 5-30 days old. After 5 days the discs were weighted again and the average weight of eaten food was calculated. Values of coefficients, *A* (absolute coefficient of deterrence) and *R* (relative coefficient of deterrence) were calculated as follows:

$$A = \frac{CC - TT}{CC + TT} \cdot 100$$

$$R = \frac{C - T}{C + T} \cdot 100$$

where *CC* is the average weight of the food consumed in the control, *TT* means the average weight of the food consumed in the no-choice test, while C and T express the average weights of the food consumed in the choice test. The sum of these two coefficients (*T*) explains deterrent activity: 200-151 very good, 150-101 good, 100- 50 medium, <50 weak.

Adjuvant activity

Seeds of common lambsquarters (*Chenopodium album* L.) cornflower (*Centaurea cyanus* L.) and silky bent grass [*Apera spica-venti* (L.) P.Beauv] were planted into commercial peat-based potting material (Kronen, Cerekwica Poland) in 0.5 L plastic pots. Plants were grown in/under controlled environmental conditions: a temperature of 20 ± 2 °C, a humidity of 60%, and a photoperiod of 16/8 h day/night. Plants were watered as needed and thinned to 5 plants per pot within 2 weeks after emergence. Herbicide Henik 50 SG (nicosulfuron, 500 g kg^{-1}) was applied in dose 30 g ai ha^{-1} alone, and with IL at 0.4% v/v or with 0.75% v/v Actirob 842 EC (rapeseed oil methyl ester, 733 g L^{-1}) at the four-leaf stage (BBCH 14). Herbicide treatments were dispersed in water and applied using a moving sprayer (APORO, Poznan, Poland). Sprayer was equipped with a XR TeeJet 110 02 VP flat-fan nozzle (TeeJet Technologies, Wheaton, IL, USA) delivering 200 L ha^{-1} spray mixture at 200 kPa operating pressure. Fresh weight of plants was measured three weeks after treatment using a technical balance with 0.01 g accuracy (Sartorius BP 2000 S, Sartorius Göttingnen, Germany). The efficacy data is expressed as percent fresh weight reduction compared with control (non-treated plants). Each experiment was conducted as a completely randomized design with four replications. The data concerning weed control were subjected to the analysis of variance, in case of weed control check plots were excluded using Tukey' test, determining the highest significant difference on the level of significance 5% (HSD).

(1) Stuper-Szablewska, K.; Buśko, M.; Góral, T.; Perkowski, J. The fatty acid profile in different wheat cultivars depending on the level of contamination with microscopic fungi. *Food Chem.* **2014***, 153*, 216–223.

(2) Shaihidi, F.; Wanasundara, N. In: Chi-Tang, H.; Chee-Teck, T.; Chao-Hsiang, T. (ed). *Flavor Technology: Physical Chemistry, Modification and Process*, American Chemical Society (ACS Symposium Series No. 610), Washington DC, pp 139-151, 1995.

(3) Nielsen, S.S. *Food Analysis*, 2nd ed., Aspen Publishers, Inc.: Gaithersburg, MD, 1998.

(4) Klejdysz, T.; Łęgosz, B.; Czuryszkiewicz, D.; Czerniak, K.; Pernak J. Bio-based ionic liquids with abietate anion. *ACS Sustainable Chem. Eng.* **2016**, *4*, 6543–6550.

(5) Furniss, B. S.; Hannaford, A. J.; Smith, P. W. G.; Tatchell, A. R. Vogel`s Textbook of Practical Organic Chemistry, 5th ed., Longman Scientific & Technical, John Wiley & Sons, New York, 1989.
(6) Szczepanik, M. Obara, R. Szumny, A., Gabryś, B. Halarewicz-Pacan, A. Nawrot, J. Wawrzeńczyk, C. Synthesis and insect antifeedant activity of precocene derivatives with lactone moiety. *J. Agric. Food Chem.* **2005**, 53, 5905-5910.
